# Healthcare Quality Improvement and ‘work engagement’; concluding results from a national, longitudinal, cross-sectional study of the ‘Productive Ward-Releasing Time to Care’ Programme

**DOI:** 10.1186/s12913-017-2446-2

**Published:** 2017-08-01

**Authors:** Mark White, Tony Butterworth, John SG Wells

**Affiliations:** 1 Director, Nursing and Midwifery Planning and Development Unit, Office Complex, Kilcreene Hospital, Kilkenny, R95 DK07 Ireland; 2 Emeritus Professor of Healthcare Workforce Innovation, University of Lincoln, Lincoln, UK; 3 Head of School of Health Science, Waterford Institute of Technology, Waterford, Ireland

**Keywords:** Employee engagement, Health service research, Hospital units, Lean healthcare, Multidisciplinary care team, Nurse attitudes, Productive ward, Program implementation, Quality improvement

## Abstract

**Background:**

Concerns about patient safety and reducing harm have led to a particular focus on initiatives that improve healthcare quality. However Quality Improvement (QI) initiatives have in the past typically faltered because they fail to fully engage healthcare professionals, resulting in apathy and resistance amongst this group of key stakeholders. Productive Ward: Releasing Time to Care (PW) is a ward-based QI programme created to help ward-based teams redesign and streamline the way that they work; leaving more time to care for patients. PW is designed to engage and empower ward-based teams to improve the safety, quality and delivery of care.

**Methods:**

The main objective of this study was to explore whether PW sustains the ‘engagement’ of ward-based teams by examining the longitudinal effect that the national QI programme had on the ‘work-engagement’ of ward-based teams in Ireland. Utilising the Utrecht Work Engagement Scale questionnaire (UWES-17), we surveyed nine PW (intervention) sites from typical acute Medical/Surgical, Rehabilitation and Elderly services (representing the entire cohort of a national phase of PW implementation in Ireland) and a cohort of matched control sites. The numbers surveyed from the PW group at T1 (up to 3 months after commencing the programme) totalled 253 ward-team members and 249 from the control group. At T2 (12 months later), the survey was repeated with 233 ward-team members from the PW sites and 236 from the control group.

**Results:**

Overall findings demonstrated that those involved in the QI initiative had higher ‘engagement’ scores at T1 and T2 in comparison to the control group. Total ‘engagement’ score (TES), and its 3 dimensions, were all significantly higher in the PW group at T1, but only the Vigour dimension remained significantly higher at T2 (*p* = 0.006).

**Conclusion:**

Our results lend some support to the assertions of the PW initiative itself and suggest that when compared to a control group, ward-based teams involved in the QI programme are more likely to be ‘engaged’ by it and its associated improvement activities and that this is maintained over time. However, only the Vigour dimension of ‘engagement’ remained significantly higher in the PW over time.

**Electronic supplementary material:**

The online version of this article (doi:10.1186/s12913-017-2446-2) contains supplementary material, which is available to authorized users.

## Background

There are many drivers for improving quality in healthcare. The pressures associated with growing populations, changing healthcare needs, increasing healthcare costs, in addition to concerns about patient safety and reducing harm are amongst some of the most compelling. In addition, poor perception and experiences of healthcare from both patients and the media have collectively combined to provide many of the ingredients for a ‘perfect storm’ in healthcare crisis terms. This has led to an international call-to-action to ‘rescue’ the provision of healthcare with a particular renewed focus on initiatives that improve the quality of healthcare [1, 2].

Although the requirement to improve healthcare whilst trying to master what works well and why is very well established; there is in fact a limited understanding of the exact impacts, outcomes and outputs from many of the interventions that are designed to improve healthcare quality. This lack of understanding has created an interest amongst researchers and clinicians to articulate what constitutes a successful healthcare QI intervention or initiative [3].

Some of the current views on implementing healthcare quality innovations have their foundational basis in Pressman and Wildavsky’s study of policy implementation, [4] and Havelock’s change agent studies in education [5]. There appears to be broad agreement in the literature that the implementation of healthcare QI is decidedly more complex and fraught with many more variables than had been previously assumed [6, 7].

In this regard, what is termed ‘Improvement Science’ has evolved to look beyond the descriptive theories of innovation, implementation and change and to focus on other important components that are required for effective implementation of QI interventions and strategies. These include the many contextual variables such as circumstances, behaviours and interactions that result in improved quality.

QI initiatives have in the past typically failed to ‘engage’ healthcare professionals and faltered as a result. Many studies, for example, report apathy and resistance from clinicians when such initiatives are introduced [8, 9]. In this regard, getting healthcare professionals to think and behave in different ways is not all that straightforward and many need to be convinced of the value and merits of improvement methods tools or programmes [10]. For some healthcare professionals QI can be perceived as a ‘job-demand’ that detracts from clinical care and not a ‘resource’ that enables them to improve quality [11]. It is now widely acknowledged that engaging clinicians (regardless of setting or discipline) is a precondition for the success of QI initiatives [12, 13].

In trying to find a panacea for what ‘engages’ healthcare professionals effectively, many healthcare organisations have attempted to replicate the improvement and ‘engagement’ successes lauded in the manufacturing sector. QI programmes adopted from industry and remodelled or adapted for the health care setting currently take a variety of formats, including ‘Lean Healthcare’, ‘Six Sigma’, ‘Clinical Microsystems’ and the ‘Model for Improvement’.

It could be argued that many of these programmatic approaches to healthcare QI have also failed to fully ‘engage’ healthcare employees and that this may in part, be due to the bifurcation of intent between the significant stakeholders upon whom healthcare QI success depends [10]. Healthcare professionals’ views and understanding of QI have been shown to be divergent from that of managers [14]. Indeed, clinicians can often perceive these initiatives as a threat to their professional practice, identities, status and power [15].

This dichotomy of world views has been previously described in terms of competing logics - professional, managerial and institutional [16, 17], and provides some explanation as to why many front-line clinical staff (including nurses) generally steer away from the financial aspects of care and direct their attention and efforts towards care provision and the patient experience [18]. It also provides some understanding as to why non-clinical stakeholders generally tend to focus on just the one dimension; reducing the per capita cost of health care.

The challenge for contemporary healthcare QI initiatives are how they ‘engage’ the many competing logics, interests and intentions to deliver improvements in the key areas of population health, patient experience and cost [1, 18]. Productive Ward: Releasing Time to Care™ (PW) is one such QI programme that was designed to synergise the multiple aspects of QI and directly impact care provision, patient experience and costs [19, 20].

PW, designed and developed by the UK’s National Health Service Institute for Innovation and Improvement (NHSI) in 2005, may be best described as a ward-based QI programme which helps ward-based teams redesign and streamline the way they work in order to leave more time to care for patients. Using Lean improvement techniques, the intrinsic motivators of social movement theory and the front-line ‘engagement’ theories of large-scale change, the PW programme is designed to ‘engage’ and ‘empower’ teams to improve the safety, quality and delivery of care. The PW programme, which has been adopted in a number of countries, has been the focus of multiple studies and evaluations [21] and is reported to ‘engage’ front-line teams and clinicans in QI and QI activities [19, 20, 22].

In December 2010 Ireland made PW one of its national clinical care programmes and priorities. The recruitment of appropriate sites for implementation began in September 2011 and organisations/hospitals who had expressed an interest in participating were interviewed. A readiness audit tool (based on a previous NHSI site-selection tool) was developed to ensure that only well-prepared, well-supported sites were chosen for the initial phase. Fifty-four sites expressed an interest and in November 2011, 17 pilot sites (involving 24 wards) throughout Ireland were selected for the first phase of a national implementation.

In November 2012, a second cohort, consisting of nine wards (seven sites), were chosen for the next phase of implementation in early 2013. Evaluation of the initiative was carried out with this second implementation phase and focused on examining the ‘engagement’ claims of the QI initiative and the impact that the PW programme and its associated QI activities had on the ‘engagement’ (and the sustaining of ‘engagement’) of participants. Initial ‘engagement’ scores were obtained from this cohort in early 2013 and have been previously reported [22].

## Methods

### Objectives

The main objective of this study was to explore whether PW sustains the ‘engagement’ of ward-based teams by examining the longitudinal effect that a national QI programme (PW) had on ‘engaging’ ward teams by:

- Measuring and comparing the preliminary ‘engagement’ scores from the ward-based teams involved in the national pilot phase of the PW and a control group (of similar size, from similar clinical specialty areas, who were not involved in a quality improvement programme, initiative or improvement activity) [22], as a baseline measure (T1).
- Measuring and comparing ‘engagement’ scores within both the intervention and control group again approximately 12 months later (T2).
- Comparing changes in ‘engagement’ scores (T2-T1) in the intervention and control groups, controlling for other variables.

### Study design

This study uses a longitudinal cohort survey design to examine ward team ‘engagement’ (nursing and non-nursing) in the national ‘PW’ QI initiative in Ireland across two time periods. Ethics approval was provided by Waterford Institute of Technology and the Health Service Executive.

### Setting

Nine PW (intervention) sites (representing the entire cohort of a national phase of PW implementation in Ireland) and a cohort of matched control sites participated in this study. The nine PW sites represent the typical acute Medical/Surgical, Rehabilitation and Elderly services that participated in the entire national PW initiative (see Table 1). All resident ward team members (nursing and non-nursing) were invited to participate in this study.

**Table 1:** Research setting and response rates

PW Site	1	2	3	4	5	6	7	8	9	Total
Clinical Specialty	Rehab	Elderly	Surge	Surge	Med	Med	Med	Elderly-Rehab	Med	-
Numbers Surveyed N =	20	45	38	27	25	19	28	24	27	**253**
T1 Response Rate N =	17 (85%)	43 (95.5%)	23 (60.5%)	22 (81.4%)	9 (36%)	13 (68.4%)	19 (65.5%)	17 (70.8%)	21 (88.8%)	**180 (71.1%)**
T2 Response Rate N =	13 (65%)	25 (91.3%)	24 (63.1%)	17 (62.9%)	22 (88%)	7 (36.8%)	20 (68.9%)	17 (70.8%)	24 (88.8%)	**169 (66.8%)**
T1 & T2 Response Rate N =	10 (50%)	24 (53.3%)	13 (34.2%)	13 (48.1%)	4 (16%)	5 (26.3%)	9 (32.1%)	10 (41.6%)	13 (48.1%)	**101 (39.9%)**
Control Site	***A***	***B***	***C***	***D***	***E***	***F***	***G***	***H***	***I***	***Total***
Clinical Specialty	*Rehab*	*Elderly*	*Surge*	*Surge*	*Med*	*Med*	*Med*	*Elderly-Rehab*	*Med*	*-*
Numbers Surveyed N =	*20*	*42*	*35*	*26*	*24*	*26*	*26*	*24*	*26*	***249***
T1 Response Rate N =	*18 (90%)*	*18 (42.8%)*	*19 (54.3%)*	*20 (77%)*	*14 (58.3%)*	*22 (84.6%)*	*18 (69.2%)*	*15 (62.5%)*	*14 (53.8%)*	***158 (63.4%)***
T2 Response Rate N =	*18 (90%)*	*14 (33.3%)*	*20 (57.1%)*	*17 (65.3%)*	*8 (33.3%)*	*26 (100%)*	*20 (76.9%)*	*21 (87.5%)*	*17 (65.3%)*	***161 (64.6%)***
T1 & T2 Response Rate N=	*15 (75%)*	*13 (31%)*	*5 (14.3%)*	*7 (27%)*	*6 (25%)*	*13 (50%)*	*13 (50%)*	*9 (37.5%)*	*10 (38.4%)*	***91 (36.5%)***

### Participants

A stratified sample of 253 ward-team members from the nine wards/units involved in the initiative (the total eligible population of a national phase of PW implementation) were identified through the project lead in each setting and surveyed in early 2013 (T1). Data was collected up to 12 weeks into the implementation of the QI programme and compared to data from a matched (approximate fit) control group collected at the same time.

The matching control quota sample (using approximate matching criteria) were recruited by local Quality leads and used as a comparator for the PW sites (see Table 1). The stratification characteristics of the control group - a purposive sample - were:

- number of wards/units (*n* = 9)
- similar clinical specialty/ward environment
- similar ward size and sample size
- consent to participate in the study
- non-participation in a QI initiative

Table 2 below, shows the grade and age distributions of the respondent sample (T1) and highlights the strong relationship between age and employment grade. For example, it is seen that the Staff Nurse grade comprises 100% of the 18–24 age group, 72.2% of the 25–44 age group, but only 53.1% of the 45–65 age group. The vast majority of respondents were in either the 25–44 age group (57.7%) or the 45–65 age group (38.7%).

**Table 2:** Age and grade distribution of respondents

			Grade					Total
			Nurse Manager	Staff Nurse	Clerical/Admin	Care Assistant/MT Attendant	Household	
**Age**	**18–24 years**	Count	0	13	0	0	0	13
		% within age	0.0%	100.0%	0.0%	0.0%	0.0%	100.0%
	**25–44 years**	Count	14	140	4	35	1	194
		% within age	7.2%	72.2%	2.1%	18.0%	0.5%	100.0%
	**45–65 years**	Count	15	70	8	34	4	131
		% within age	11.5%	53.1%	6.2%	26.2%	3.1%	100.0%
**Total**		**Count**	**29**	**223**	**12**	**69**	**5**	**338**
		**% within age**	**8.6%**	**65.8%**	**3.6%**	**20.5%**	**1.5%**	**100.0%**

Because of this strong relationship between employment grade and age, we did not control for age in subsequent analyses. Neither did we control for gender, as the sample was overwhelmingly female. The variables analysed in this study were, therefore, ‘engagement’ scores, changes in these ‘engagement’ scores over time, employment grade, specialty and group (PW/control).

#### Description sample T2

This phase of the research design was a repeat of the T1 survey (same intervention, PW sites, control group, instrument and procedure) approximately 12 months later. At T2, 233 ward-team members from the PW sites and 236 from the control group were again surveyed. A descriptive breakdown of T2 participants is included in Table 1 and Table 3. Staff Nurse grades again represented the largest group of respondents (68.2%) in the sample, followed by Healthcare Support workers (16.1%) and Nurse Managers (8.6%).

**Table 3:** Descriptive breakdown of participants who completed both T1 & T2

	PW Group	*Percent*	*Control Group*	*Percent*	Total	*Percent*
No. Surveyed T2	233	*100%*	*236*	*100%*	**469**	***100%***
No. Respondents T2	169	*72.5%*	*161*	*68.2%*	**330**	***70.4%***
No. Respondents T1 & T2	101	*52.6%*	*91*	*47.4%*	**192**	***100%***
Female	97	*96%*	*81*	*89%*	**178**	***92.7%***
Male	4	*4%*	*10*	*11%*	**14**	***7.3%***
Age: 18–24	4	*4%*	*2*	*2.2%*	**6**	***4.6%***
Age 25-44	56	*55.4%*	*52*	*57.1%*	**108**	***60.7%***
Age 45-65	41	*40.6%*	*37*	*40.7%*	**78**	***34.7%***
Nurse Managers	9	*8.9%*	*16*	*17.6%*	**25**	***13%***
Staff Nurses	70	*69.3%*	*61*	*67%*	**131**	***68.2%***
Clerical/Admin	1	*1%*	*2*	*2.2%*	**3**	***1.6%***
Healthcare Support	20	*19.8%*	*11*	*12.1%*	**31**	***16.1%***
Household	1	1%	*1*	*1.1%*	**2**	**1%**

### Data sources/measurement

The 17-item Utrecht Work Engagement Scale questionnaire (UWES-17) -a three-dimensional model of vigour, dedication and absorption-, [23] was used to measure the total levels of ‘engagement’ (TES). The measure conceptualises ‘engagement’ as a positive, fulfilling, work-related state of mind and is characterised by positive combinations of all three dimensions.

- Vigour refers to high levels of energy and mental resilience while working; the willingness to invest effort in one’s work and persistence even in the face of difficulties
- Dedication refers to being strongly involved in one’s work and experiencing a sense of significance, enthusiasm, inspiration, pride, and challenge
- Absorption refers to being fully concentrated and happily engrossed in one’s work whereby time passes quickly and one has difficulties with detaching oneself from work [24].

The concept and measure is grounded in the job-demands domain of the job demands/job-resources conceptual framework where job-resources are positively valued physical, social or organisational aspects of the job that are integral to achieving work goals or reduce job-demands (negatively valued physical, social, or organisational aspects of the job) [25].

Each item is scored on a seven-point rating scale from 0 (never) to 7 (every day). The mean scale score (TES) of the three UWES subscales is computed by adding the scores on the particular scale and dividing the sum by the number of items of the subscale involved. A similar procedure is followed for the total score. Hence, the UWES, yields three subscale scores and/or a total score (TES) that range between 0 and 6.

The UWES is the most established and widely accepted measure of employee ‘engagement’ in the academic literature, [26, 27] and has repeatedly been shown to be psychometrically sound and reliable in a variety of international healthcare settings [28–30].

### Statistical methods

Data were analysed using the commercial software SPSS (version 21). Frequency and descriptive statistics were generated for each of the variables contained in the questionnaire. Statistical analyses which we had performed included:

- Standard reliability analysis of the questionnaire items, in order to confirm suitability of the UWES-17 scales in both a QI and Irish setting;
- Comparison of UWES scores (total ‘engagement’ scores and the individual constructs) in PW and control groups, using independent sample t-tests;
- Investigation of relationships between ‘engagement’ scores and other variables, using t-tests or contingency table analysis, as appropriate;
- Analysis (using general linear models) of ‘engagement’ scores in PW and control groups, controlling for confounding variables identified in (c);

Non-parametric analogues of t-tests were also employed, as appropriate, e.g. when ‘engagement’ scores were found to be not normally distributed. Five percent level of statistical significance was adopted throughout this study, without adjustment for multiple testing.

For the current study we changed the general linear model analysis to a linear mixed model analysis, following a journal reviewer’s recommendation that the analysis should reflect that study participants were clustered in Wards. We repeated the earlier T1 analysis of ‘engagement’ scores, using the new models, and continued to use the new models for the analysis of T2 ‘engagement’ score’s, and for the analysis of change in ‘engagement’ scores from T1 to T2. In all cases, the models chosen had Ward as a Level 2 variable, a random intercept term, and explanatory variables Group (PW/control), employment grade and specialty. Only main effect models were analysed.

## Results

### T1

The initial results obtained at T1 have been reported previously, the main finding being that, controlling for employment grade and specialty, all ‘engagement’ scores at T1 were significantly higher in the PW group compared to the control group [22]. However, that conclusion was based on general linear model analyses, and on a larger sample than the current study. In the current study, we repeated the T1 analyses of ‘engagement scores’, based on subjects who also completed study questionnaires at T2, and on this occasion (as suggested by a journal reviewer) we used linear mixed model analysis (with Ward as a Level 2 variable) instead of the general linear models used in the earlier analysis.

We now report that the main conclusions from the earlier study still stand. Based on the linear mixed model analyses, and the smaller sample size, TES at T1, and its separate components, are all significantly higher in the PW group. Table e (Additional file 1) has detailed output from the mixed models; Table 4 (upper panel) below, summarises the main T1 findings from these models, as they relate to ‘engagement’ scores – we present effect sizes (average amount by which PW ‘engagement’ scores exceed control ‘engagement’ scores), and 95% confidence intervals (CI’s) for each effect. For all ‘engagement’ measures, the effects are positive, substantial and statistically significant i.e. all ‘engagement’ scores are significantly higher, on average, in the PW group compared to the control group.

**Table 4:** T1 & T2 effect sizes and *p*-values

*Total Sample N = 192*				
Time	Engagement Score	Estimated Effect size PW V’s Control	95% CI	*p*-value
T1	TES	0.36	(0.09, 0.63)	0.009
	Vigour	0.42	(0.12, 0.72)	0.006
	Absorption	0.44	(0.12,0.76)	0.007
	Dedication	0.43	(0.11, 0.74)	0.008
T2	TES	0.22	(−0.03, 0.46)	0.079
	Vigour	0.38	(0.11, 0.65)	0.006
	Absorption	0.08	(−0.20,0.36)	0.569
	Dedication	0.19	(−0.09, 0.48)	0.184

Also Included in table e (Additional file 1), but not in Table 4 (which focuses on ‘engagement’), are some interesting details about the other explanatory variables, employment grade and specialty. There are statistically significant differences among employment grades, in TES, vigour and absorption at T1, with Nurse Managers consistently showing higher ‘engagement’ scores than other categories e.g. from the mixed model output, it is seen that the TES at T1 for Nurse Managers is 0.36 higher than for support staff and 0.67 higher than for staff nurses. Elderly specialties had consistently higher T1 ‘engagement’ scores than other specialties, but these were not statistically significant.

### T2

In total, 192 participants completed and returned surveys in both T1 and T2 phases, representing a total response rate of 56.8% of the original 338 participants who returned surveys in T1, but only 38.2% of the 502 originally surveyed 12 months previously (Table 3). At T2, 101 responded from the PW group (52.6%) and 91 responded from the control group (47.4%). The total 192 completed questionnaires were included in the T1/T2 analysis reported below, but in analyses involving employment grades, only 187 subjects were included, because we removed the two employment grades with small numbers (Clerical and Household).

#### 'Engagement' scores in PW and control groups at T2 T2

**Fig. 1:**
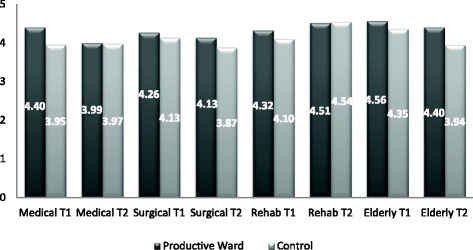
T1 & T2 ‘Engagement’ Scores by clinical specialty; highlighting the higher engagement scores that were generally maintained in the PW group over the two time periods

We used the same statistical approach (linear mixed models) as described above, with T2 ‘engagement’ scores replacing the T1 scores as dependent variable in each analysis. Table’s m and n (Additional file 1) have the detailed output from these models. Table 4 (lower panel) and Fig. 1 below highlights the main findings as they relate to comparison of PW and control groups. In general, following these analyses, T1 results were replicated at T2 and the overall conclusions remain the same: the effect sizes in Table 4 (lower panel, T2) above, are all still positive (after controlling for clinical specialty and employment grade), meaning that T2 ‘engagement’ scores were still uniformly higher in the PW group when compared with the control group; however, most of these PW scores were no longer significantly higher (at the 5% level) at T2.

Table n (Additional file 1) also has information (from the T2 mixed models) on the relationship of T2 ‘engagement’ scores to specialty and employment grade. It is seen that the superiority of ‘engagement’ scores in the Nurse Manager grade continues at T2 (although not always significantly higher). The main difference is that, at T2, statistically significant differences among specialties becomes evident, with the Rehab specialty now showing substantially higher ‘engagement’ scores (for vigour and dedication) than the Elderly specialty (which had uniformly higher scores at T1).

#### Changes in ‘engagement’ scores from T1 to T2

It is evident, from the T1 and T2 findings reported above, that statistically significant results at T1 were not always replicated at T2. For the remainder of this results section, we provide information on some of the changes that occurred between T1 and T2, which may have contributed to the observed discrepancies in the two sets of results.

Table 5 presents the average ‘engagement’ scores (total and individual dimensions) at both of T1 and T2, separately within each group, and (final column) the independent t-test *p* values for between-group comparison of changes in these scores over the study period. As seen in Table 5, absorption score grew significantly more (over the 12-month period) in the control group, explaining why there was no longer a statistically significant difference in absorption between groups at T2. Changes in the other three ‘engagement’ scores were not significantly different between groups, but in all three cases the PW TES declined from T1 to T2, whereas the control group TES and dedication scores increased. Table 4, also highlights that only vigour remained significantly higher in the PW group at T2.

**Table 5:** T1 & T2 means, standard deviations and *p* values for change between T1 and T2

*Total Sample N = 192*			
	PW Group	*Control Group*	*P* value for comparing change between groups
T1 & T2 N=	101	*91*	-
TES T1	4.39	*4.07*	
SD ±	0.82	*0.99*	0.154
TES T2	4.23	*4.10*	
SD ±	0.85	*0.88*	
Vigour T1	4.24	*3.88*	
SD ±	*0.92*	*1.11*	0.454
Vigour T2	*4.11*	*3.88*	
SD ±	*0.92*	*0.99*	
Absorption T1	4.08	*3.70*	
SD ±	1.03	*1.13*	0.022
Absorption T2	4.09	*4.06*	
SD ±	1.04	*0.88*	
Dedication T1	4.74	*4.35*	
*SD ±*	0.85	*1.22*	0.071
Dedication T2	4.53	*4.42*	
SD ±	0.95	*1.06*	

We also used a linear mixed model, with T2-T1 difference as dependent variable, specialty, grade and group (PW, Control) as factors, and Ward as a Level II variable (see Additional file 1: Tables y,x,u). These analyses revealed (i) that the T2-T1 change in absorption score was significantly different between PW and control groups (*p* = 0.024 in linear mixed model, with control group showing the greater increase Additional file 1: Table y) – confirming the finding from the simpler analysis in Table 5 and (ii) that T2-T1 change in TES (*p* = 0.013) and vigour (*p* = 0.011) were significantly different by specialty (Additional file 1: Tables x,u) – with Rehab showing the greatest improvement on both scores. Apart from these, T2 – T1 changes in ‘engagement’ scores were not significantly different from changes in the control group ‘engagement’ scores.

## Discussion

### Key results

The primary aim of this study was to examine the longitudinal impact of the nationally introduced QI initiative PW in Ireland on the levels of ‘engagement’ in the ward teams implementing it and to establish whether they were more ‘engaged’ than ward teams not involved in PW or any other QI intervention. The findings of this study only partially confirm the previous findings [22] at T1, in that ‘engagement’ scores, controlling for employment grade and clinical specialty, remained higher at T2 in the group implementing the PW initiative compared to a control group. However, only the Vigour score was significantly higher in the PW group at T2 (*p* = 0.006).

At a time when it is becoming widely acknowledged that engaging clinicians from all settings and disciplines is a precondition for the success of QI initiatives, [12, 13, 31] the findings from this study provide mildly encouraging evidence that PW, as a programmatic QI approach, is quite likely to ‘engage’ its participants and sustain moderate levels of interest, energy (vigour) and ‘engagement’. This may in part be due to the initial testing, piloting, marketing and investment that went into its development. It has previously been reported that the ‘releasing time to care’ strapline was added pre-launch, following initial piloting, to further ‘engage’ and ‘entice’ nurses and ward teams who had declared that the QI programme represented a hard industrial methods approach [32]. The use of marketing and straplines that connect healthcare QI implementers to a ‘purpose’ has been previously described within the ‘social movement theory’ context and outlined as a critical enabler for QI ‘engagement’ and success [33].

The ‘connection to a purpose’ approach adopted by PW has however received some criticism, for creating a state of ‘desirability’ amongst nurses, [34] where it has been argued that nurses are potentially drawn to ‘engage’ with the programme on a professional ‘principle’ basis (releasing time to care) and not because improving all aspects of healthcare quality is the right thing to do. Sustaining ‘engagement’ in PW on this professional principle-basis would be a most difficult endeavour. Mostly because the purpose ‘releasing time to care’ has been difficult to definitively measure and improve [32]. The modestly positive ‘engagement’ findings (compared to a control group) across two time periods, would however suggest that to some extent, the PW programme does not just ‘engage’ ward-based teams on an initial principle-based or social movement basis, it is quite possible that it can continue to ‘engage’ ward team members (in the the various QI activities) over a prolonged (12-month) period.

Previous reports of PW, [20, 35–37] have indicated how clinical workload; bed shortages; sick leave; increased winter activity and shortage of temporary/relief staff have affected the morale and participation of staff (hampering ‘engagement’ and progress with some QI activities). In the current study, there was some decline in the PW group ‘engagement’ scores over time, and one could argue that this may be partly have been due to the implementation of this large-scale QI programme (and study) being undertaken during the most austere of economic times in Ireland [38]. However, whilst participants involved in this national PW maintained their higher ‘engagement’ scores there were increases observed in the control group.

We believe that there are a number of possible contextual explanations for the PW group generally maintaining their higher scores including:

- the phased modular content and associated ease of learning QI methods,
- the local supports associated with the national and structured implementation in Ireland,
- the perception that this initiative was one of the few development opportunities being offered to staff during an austere-induced drought of development in healthcare in Ireland,
- the positive impact that the QI tools/methods had on participants levels of professional self-efficacy; which have been recently been identified as a predictor of ‘engagement’ [39].

It could be argued that the austerity cuts to healthcare in Ireland did have some effect or impact on the control group. A moderate rise in ‘absorption’ scores (the only statistically significant group effect detected when changes in scores from T1 to T2 were analysed) was observed. It is noteworthy though that no increases were observed in the control group in relation to the other dimensions ‘vigour’ or ‘dedication’. It could be postulated that the increased activity, short staffing, reduced promotional opportunities/training and cost-cutting that took place during the economic crisis may inadvertently have encouraged ward-based teams in the control group to just focus their attention on and be more ‘absorbed’ by their clinical work; in-line with the expression of ‘keeping your head at work’ and a possible defensive coping mechanism to combat additional ‘stress’ [40].

The moderately higher TES’s for the non-nursing Clerical/Administration and Household (indirect care) team members that were observed at T1 in both the PW and control groups, could not be explored further as a result of reduced participants at T2. The findings from T1 however should not be ignored. Higher levels of stress and emotional demands are regularly reported in many healthcare occupations, like nursing (the predominant employment grade in this study), who provide direct patient care. This has been shown to make this group particularly susceptible to burnout, [40–44] which is well recognised as an antithesis to vigour (high levels of energy and mental resilience while working) and ‘engagement’ [45–47]. This offers some explanation as to why the nursing employment grades recorded slightly lower ‘engagement’ scores than ward-team members not involved in direct patient care and this should be considered in further measures of ‘engagement’ within the QI arena.

The significantly higher TES’s at T1 for the Elderly sites were much more moderate at T2. However the elevated TES’s that were observed in the other non-acute setting Rehab, remained elevated in both the PW and the control group. Indeed this non-acute setting represented the only clinical site to significantly improve their ‘engagement’ scores over the two time periods. Whilst some studies have shown different levels of ‘engagement’ between the acute and non-acute sectors, [48] this study is amongst the first to demonstrate higher levels of ‘engagement’ in non-acute settings involved in a programmatic QI intervention and merits further investigation.

This may in part be due to the very contrasting contexts, competing commitments to QI systems and processes experienced between the acute and non-acute healthcare settings [49].

It also may be due to lower levels of burnout reported in many non-acute settings that are a result of contrasting work-related factors [42, 50], (reduced patient turnover; a reduced focus on budgets and targets; higher levels of organisational slack and more of a focus on person-centeredness) that are not observed in the busy acute healthcare sector.

The findings from this study provide some evidence that ‘off the shelf’ programmatic QI initiatives like PW are most likely to be perceived as a ‘job-resource’ and have the capacity to ‘engage’ and maintain that ‘engagement’ for at least 12 months. It also adds to the growing body of knowledge of ‘engagement’ and the job-demands/job-resources conceptual framework within a QI context and further validates the use of the ‘work engagement’ construct and measure as being suitable for use with healthcare QI.

Finally this study provides some response to the critics of lean-based QI programmes like PW, [51, 52] who have raised doubts about the impact and sustainability of such initiatives and what they can meaningfully deliver. If ‘engagement’ is one of the key challenges to improving quality, [9, 13] the modestly positive, sustained results from the PW initiative in Ireland (particularly around the vigour dimension) are encouraging.

### Bias

Potential biases can be identified in the national implementation strategy employed, roll-out scheduling, as well as the selection of clinical units involved in both phases of PW. We sought to avoid an additional source of bias by the use of anonymous subject ID’s when collecting and recording responses from individual study participants.

### Limitations

Firstly, it is important to highlight that the PW (intervention group) were selected as part of a national pilot phase of the initiative. It is plausible that many of the organisations and wards who put themselves forward for the pilot have some degree of highly motivated individuals who applied or have a track record as a high performing ward or department.

A second limitation of this study is the use of a non-probability quota sampling strategy for recruiting the control group. Whilst the characteristics of size and clinical context of the control group generally reflect that of the PW group, the sample is in essence a purposive sample; the matching exercise, no matter how rigorous, can never be truly representative. Access to a randomised control group would of course be ‘gold standard’ for a QI study of this nature, but realistically would be extremely challenging from a number of perspectives. This study did control (using linear mixed models) for variables such as specialty and employment grade which differed between the intervention and control groups, and which were also related to the ‘engagement’ outcome measures.

All study variables were extracted from the resident ward teams’ reported perspectives. Other objective variables and perspectives from extended ward-team members (visiting medical colleagues and allied healthcare professionals) could extend confirmation of these study results.

Finally, although this study examines the ‘engagement’ scores across two time points, it could be considerably strengthened with pre-intervention measures and additional data from further phases which would allow more complex analysis of relationships between employment grade, specialty site and the control group. It would also go some way to help address the growing concerns in regard to understanding the many influences involved in sustaining healthcare QI over time [1, 53, 54]. However, it is worth noting that QI programmes, interventions and activities by their very nature are action-orientated occurrences and do not naturally lend themselves to the criteria and standards of experimental design. Pre-intervention measures were hampered in this study by long delays in ethics approval and the overriding requirements by the services to start the QI initiative and associated activities.

### Generalisability

This study was performed across a variety of acute and non-acute hospital settings in one national health system and generalisation to other settings should be approached with caution. All findings in this study can only be viewed through the lens in which they were studied, i.e. teams involved in implementing the PW in Ireland. However, the generalisability and transferability of learning from all QI initiatives deserves consideration when trying to broaden, spread or replicate QI efforts as there are many organisational, contextual and social challenges to take into account [55, 56].

## Conclusion

Overall these results suggest that when compared to a control group (of similar size, from similar clinical specialty areas, who were not involved in a quality improvement programme, initiative or improvement activity) ward-based teams who participate in the QI programme PW are more likely to be ‘engaged’ by it and its associated improvement activities and it is possible to maintain these elevated levels of ‘engagement’ over a 12-month period. Further longitudinal studies with pre-intervention measures are required to examine how sustained this effect might be. We hypothesise that the relatively stable positive ‘engagement’ measures observed in this study indicate that PW and its QI activities are most likely viewed as a job-resource (a positive, valued aspect of one’s job which may also possibly negate the job-demands of one’s job). However, using the job demands/job resources theoretical framework within a healthcare QI context requires further exploration and comparison with and against other QI initiatives. This study demonstrates the complexity of QI implementation and that ‘one size does not fit all’, by reporting the variances in ‘engagement’ scores across different clinical settings and various employment grades. It raises the question that there may be certain components of the PW programme which are more effective or have different impacts depending on each individual context, environment and implementation strategy and these merit further exploration. Practically these findings add value for healthcare QI researchers and practitioners by endorsing (to some degree) the ‘engagement’ claims of PW and its developers. They also provide support for the concept, content and implementation approach of PW as a lean-based, programmatic approach to engaging ward-based teams and improving quality.

## Additional file

Additional file 1: SPSS output for Healthcare QI and 'Work Engagement'. Linear mixed model analysis outputs. Frequency & Descriptive statistics: Tables a-d, T1 Results: Tables e-l, T2 Results: Tables m-t, T2-T2 Results: Tables u-z2.This file contains the SPSS outputs for the linear mixed model analysis performed on Total Engagement Scores, Vigour Scores, Absorption Scores and Dedication Scores at T1, T2 and T2-T1.

## Abbreviations

PW: Productive ward – releasing time to care
QI: Quality Improvement
T1: Time one
T2: Time two
TES: Total engagement score
UWES 17: 17-item Utrecht work engagement survey
