# Supplementary material for: Healthcare Quality Improvement and ‘work engagement’; concluding results from a national, longitudinal, cross-sectional study of the ‘Productive Ward-Releasing Time to Care’ Programme
Source: BMC Health Serv Res. 2017 Aug 1;17:510. doi: 10.1186/s12913-017-2446-2 (PMC5540515; doi:10.1186/s12913-017-2446-2)
Supplement: Additional file 1: — SPSS output for Healthcare QI and 'Work Engagement'. Linear mixed model analysis outputs. Frequency & Descriptive statistics: Tables a-d, T1 Results: Tables e-l, T2 Results: Tables m-t, T2-T2 Results: Tables u-z2.This file contains the SPSS outputs for the linear mixed model analysis performed on Total Engagement Scores, Vigour Scores, Absorption Scores and Dedication Scores at T1, T2 and T2-T1. [file 12913_2017_2446_MOESM1_ESM.docx]

**SPSS output: Healthcare Quality Improvement and ‘work engagement’; concluding results from a national, longitudinal, cross-sectional study of the ‘Productive Ward-Releasing time to care’ programme.**

| **Table a: Specialty** | | | | | |
| --- | --- | --- | --- | --- | --- |
|  | | Frequency | Percent | Valid Percent | Cumulative Percent |
|  | Medical | 73 | 39.0 | 39.0 | 39.0 |
|  | Surgical | 36 | 19.3 | 19.3 | 58.3 |
|  | Rehab | 42 | 22.5 | 22.5 | 80.7 |
|  | Elderly | 36 | 19.3 | 19.3 | 100.0 |
|  | Total | 187 | 100.0 | 100.0 |  |

| **Table b: Group** | | | | | |
| --- | --- | --- | --- | --- | --- |
|  | | Frequency | Percent | Valid Percent | Cumulative Percent |
|  | PW Group | 99 | 52.9 | 52.9 | 52.9 |
|  | Control Group | 88 | 47.1 | 47.1 | 100.0 |
|  | Total | 187 | 100.0 | 100.0 |  |

| **Table c: Employment Grade** | | | | | |
| --- | --- | --- | --- | --- | --- |
|  | | Frequency | Percent | Valid Percent | Cumulative Percent |
|  | Nurse Manager | 25 | 13.4 | 13.4 | 13.4 |
|  | Staff Nurse | 131 | 70.1 | 70.1 | 83.4 |
|  | Care Assistant/ MT Attendant | 31 | 16.6 | 16.6 | 100.0 |
|  | Total | 187 | 100.0 | 100.0 |  |

| **Table d: Descriptive Statistics** | | | | | |
| --- | --- | --- | --- | --- | --- |
|  | N | Minimum | Maximum | Mean | Std. Deviation |
| Total Engagement T2 | 187 | 1.12 | 6.00 | 4.1718 | .85203 |
| VigourT2 | 187 | .17 | 6.00 | 3.9927 | .94661 |
| AbsorptionT2 | 187 | .50 | 6.00 | 4.0918 | .94470 |
| DedicationT2 | 187 | 1.40 | 6.00 | 4.4824 | .99561 |
| Change in Total Engagement Score | 187 | -2.38 | 2.96 | -.0495 | .92039 |
| Change in Vigour score | 187 | -2.83 | 3.00 | -.0575 | 1.10287 |
| Change in Absorption score | 187 | -2.17 | 3.83 | .2104 | 1.05124 |
| Change in Dedication score | 187 | -3.20 | 3.20 | -.0695 | 1.06482 |
|  | 187 |  |  |  |  |

**Mixed model results for T1**

**TES at T1**

| **Table e: Type III Tests of Fixed Effects^a^** | | | | |
| --- | --- | --- | --- | --- |
| Source | Numerator df | Denominator df | F | Sig. |
| Intercept | 1 | 180 | 2561.885 | .000 |
| Group | 1 | 180 | 7.016 | .009 |
| Specialty | 3 | 180 | .612 | .608 |
| Grade | 2 | 180 | 3.508 | .032 |
| a. Dependent Variable: TotalmeanT1. | | | | |

| **Table f: Estimates of Fixed Effects^a^** | | | | | | | |
| --- | --- | --- | --- | --- | --- | --- | --- |
| Parameter | Estimate | Std. Error | df | t | Sig. | 95% Confidence Interval | |
|  |  |  |  |  |  | Lower Bound | Upper Bound |
| Intercept | 4.289176 | .203963 | 180 | 21.029 | .000 | 3.886709 | 4.691643 |
| [Group=1] | .362231 | .136757 | 180 | 2.649 | .009 | .092378 | .632084 |
| [Group=2] | 0^b^ | 0 | . | . | . | . | . |
| [Specialty=1] | -.251677 | .208659 | 180 | -1.206 | .229 | -.663409 | .160055 |
| [Specialty=2] | -.282088 | .230377 | 180 | -1.224 | .222 | -.736676 | .172499 |
| [Specialty=3] | -.214321 | .211499 | 180 | -1.013 | .312 | -.631657 | .203014 |
| [Specialty=4] | 0^b^ | 0 | . | . | . | . | . |
| [Grade=1] | .363308 | .263296 | 180 | 1.380 | .169 | -.156236 | .882852 |
| [Grade=2] | -.153438 | .205688 | 180 | -.746 | .457 | -.559307 | .252432 |
| [Grade=4] | 0^b^ | 0 | . | . | . | . | . |
| a. Dependent Variable: TotalmeanT1. | | | | | | | |
| b. This parameter is set to zero because it is redundant. | | | | | | | |

**Comment**: For all engagement scores, the PW group was significantly higher than the control group at T1 – this confirms the earlier findings from the general linear model, and is what we published at the time. The remarkable finding now, however, is that none of the Specialty differences (see tables below) are significant at T1, and in particular, the Rehab scores at T1 are actually all lower than the Elderly scores at T1. Thus the observed superiority at T2 of the Rehab specialties was not at all evident at T1.

In short, the PW scores at T1 were all significantly better than the controls, but declined (or grew by less than the controls) over the 12 months, and only the Vigour score remained significantly higher after 12 months. The Rehab engagement scores, however, grew over the 12 months to such an extent that they outstripped the Elderly scores which had been consistently (though not significantly) better at T1.

**Mixed model results for T1**

**Vigour at T1**

| **Table g: Type III Tests of Fixed Effects^a^** | | | | |
| --- | --- | --- | --- | --- |
| Source | Numerator df | Denominator df | F | Sig. |
| Intercept | 1 | 180 | 1945.625 | .000 |
| Group | 1 | 180 | 7.649 | .006 |
| Specialty | 3 | 180 | .531 | .662 |
| Grade | 2 | 180 | 5.032 | .007 |
| a. Dependent Variable: VigourT1. | | | | |

| **Table h: Estimates of Fixed Effects^a^** | | | | | | | |
| --- | --- | --- | --- | --- | --- | --- | --- |
| Parameter | Estimate | Std. Error | df | t | Sig. | 95% Confidence Interval | |
|  |  |  |  |  |  | Lower Bound | Upper Bound |
| Intercept | 4.169053 | .227010 | 180 | 18.365 | .000 | 3.721110 | 4.616997 |
| [Group=1] | .420958 | .152210 | 180 | 2.766 | .006 | .120613 | .721303 |
| [Group=2] | 0^b^ | 0 | . | . | . | . | . |
| [Specialty=1] | -.161353 | .232236 | 180 | -.695 | .488 | -.619608 | .296903 |
| [Specialty=2] | -.292153 | .256409 | 180 | -1.139 | .256 | -.798107 | .213800 |
| [Specialty=3] | -.238416 | .235397 | 180 | -1.013 | .313 | -.702908 | .226076 |
| [Specialty=4] | 0^b^ | 0 | . | . | . | . | . |
| [Grade=1] | .356565 | .293047 | 180 | 1.217 | .225 | -.221685 | .934815 |
| [Grade=2] | -.309177 | .228930 | 180 | -1.351 | .179 | -.760908 | .142554 |
| [Grade=4] | 0^b^ | 0 | . | . | . | . | . |
| a. Dependent Variable: VigourT1. | | | | | | | |
| b. This parameter is set to zero because it is redundant. | | | | | | | |

**Mixed model results for T1**

**Absorption at T1**

| **Table i: Type III Tests of Fixed Effects^a^** | | | | |
| --- | --- | --- | --- | --- |
| Source | Numerator df | Denominator df | F | Sig. |
| Intercept | 1 | 180 | 1551.623 | .000 |
| Group | 1 | 180 | 7.431 | .007 |
| Specialty | 3 | 180 | .951 | .417 |
| Grade | 2 | 180 | 4.053 | .019 |
| a. Dependent Variable: AbsorptionT1. | | | | |

| **Table j: Estimates of Fixed Effects^a^** | | | | | | | |
| --- | --- | --- | --- | --- | --- | --- | --- |
| Parameter | Estimate | Std. Error | df | t | Sig. | 95% Confidence Interval | |
|  |  |  |  |  |  | Lower Bound | Upper Bound |
| Intercept | 3.863006 | .242460 | 180 | 15.933 | .000 | 3.384576 | 4.341437 |
| [Group=1] | .443170 | .162569 | 180 | 2.726 | .007 | .122383 | .763956 |
| [Group=2] | 0^b^ | 0 | . | . | . | . | . |
| [Specialty=1] | -.379633 | .248042 | 180 | -1.531 | .128 | -.869077 | .109811 |
| [Specialty=2] | -.416982 | .273860 | 180 | -1.523 | .130 | -.957370 | .123407 |
| [Specialty=3] | -.255299 | .251418 | 180 | -1.015 | .311 | -.751405 | .240806 |
| [Specialty=4] | 0^b^ | 0 | . | . | . | . | . |
| [Grade=1] | .639733 | .312992 | 180 | 2.044 | .042 | .022127 | 1.257338 |
| [Grade=2] | -.022688 | .244511 | 180 | -.093 | .926 | -.505164 | .459787 |
| [Grade=4] | 0^b^ | 0 | . | . | . | . | . |
| a. Dependent Variable: AbsorptionT1. | | | | | | | |
| b. This parameter is set to zero because it is redundant. | | | | | | | |

**Mixed model results for T1**

**Dedication at T1**

| **Table k: Type III Tests of Fixed Effects^a^** | | | | |
| --- | --- | --- | --- | --- |
| Source | Numerator df | Denominator df | F | Sig. |
| Intercept | 1 | 180 | 2147.313 | .000 |
| Group | 1 | 180 | 7.192 | .008 |
| Specialty | 3 | 180 | 1.540 | .206 |
| Grade | 2 | 180 | .980 | .377 |
| a. Dependent Variable: DedicationT1. | | | | |

| **Table l: Estimates of Fixed Effects^a^** | | | | | | | |
| --- | --- | --- | --- | --- | --- | --- | --- |
| Parameter | Estimate | Std. Error | df | t | Sig. | 95% Confidence Interval | |
|  |  |  |  |  |  | Lower Bound | Upper Bound |
| Intercept | 4.600370 | .237513 | 180 | 19.369 | .000 | 4.131702 | 5.069038 |
| [Group=1] | .427080 | .159252 | 180 | 2.682 | .008 | .112839 | .741321 |
| [Group=2] | 0^b^ | 0 | . | . | . | . | . |
| [Specialty=1] | -.423586 | .242981 | 180 | -1.743 | .083 | -.903044 | .055871 |
| [Specialty=2] | -.455910 | .268272 | 180 | -1.699 | .091 | -.985272 | .073452 |
| [Specialty=3] | -.121768 | .246288 | 180 | -.494 | .622 | -.607751 | .364214 |
| [Specialty=4] | 0^b^ | 0 | . | . | . | . | . |
| [Grade=1] | .276738 | .306606 | 180 | .903 | .368 | -.328266 | .881741 |
| [Grade=2] | -.044426 | .239521 | 180 | -.185 | .853 | -.517057 | .428205 |
| [Grade=4] | 0^b^ | 0 | . | . | . | . | . |
| a. Dependent Variable: DedicationT1. | | | | | | | |

**Mixed model results for T2**

**TES at T2**

| **Table m: Type III Tests of Fixed Effects^a^** | | | | |
| --- | --- | --- | --- | --- |
| Source | Numerator df | Denominator df | F | Sig. |
| Intercept | 1 | 180 | 3006.607 | .000 |
| Group | 1 | 180 | 3.117 | .079 |
| Specialty | 3 | 180 | 5.283 | .002 |
| Grade | 2 | 180 | 3.358 | .037 |
| a. Dependent Variable: Total Engagement T2. | | | | |

| **Table n: Estimates of Fixed Effects^a^** | | | | | | | |
| --- | --- | --- | --- | --- | --- | --- | --- |
| Parameter | Estimate | Std. Error | df | t | Sig. | 95% Confidence Interval | |
|  |  |  |  |  |  | Lower Bound | Upper Bound |
| Intercept | 4.096923 | .185087 | 180 | 22.135 | .000 | 3.731703 | 4.462142 |
| [Group=1] | .219093 | .124101 | 180 | 1.765 | .079 | -.025786 | .463972 |
| [Group=2] | 0^b^ | 0 | . | . | . | . | . |
| [Specialty=1] | -.368819 | .189348 | 180 | -1.948 | .053 | -.742447 | .004808 |
| [Specialty=2] | -.353726 | .209057 | 180 | -1.692 | .092 | -.766243 | .058791 |
| [Specialty=3] | .234981 | .191925 | 180 | 1.224 | .222 | -.143731 | .613693 |
| [Specialty=4] | 0^b^ | 0 | . | . | . | . | . |
| [Grade=1] | .514186 | .238929 | 180 | 2.152 | .033 | .042724 | .985648 |
| [Grade=2] | .070606 | .186652 | 180 | .378 | .706 | -.297702 | .438914 |
| [Grade=4] | 0^b^ | 0 | . | . | . | . | . |
| a. Dependent Variable: Total Engagement T2. | | | | | | | |
| b. This parameter is set to zero because it is redundant. | | | | | | | |

**Comment:** There were significant differences in TES at T2 between both Specialty’s (p=0.002) and grades (p=0.037), and nearly significant differences (P=0.079) between Groups. (Table m). Highest TES scores at T2 were observed for Rehab and Elderly specialties, Nurse Manager grades. In fact (see output tables p-t below) the three highest mean subgroup TES scores at T2, all in excess of 5) were Nurse Managers: two in the PW group, in the Elderly and Medical specialties, and one in the control group, in the Elderly specialty. The next two highest were also Nurse Managers: Elderly and Rehab, both in the control group. In fact, apart from Surgical specialties, where differences in mean scores between grades are minor, there is a consistent pattern in the table below of TES scores for Nurse Managers being substantially higher than for Staff Nurses.

**Mixed model results for T2**

**Vigour at T2**

| **Table o: Type III Tests of Fixed Effects^a^** | | | | |
| --- | --- | --- | --- | --- |
| Source | Numerator df | Denominator df | F | Sig. |
| Intercept | 1 | 180 | 2319.662 | .000 |
| Group | 1 | 180 | 7.749 | .006 |
| Specialty | 3 | 180 | 4.532 | .004 |
| Grade | 2 | 180 | 5.888 | .003 |
| a. Dependent Variable: VigourT2. | | | | |

| **Table p: Estimates of Fixed Effects^a^** | | | | | | | |
| --- | --- | --- | --- | --- | --- | --- | --- |
| Parameter | Estimate | Std. Error | df | t | Sig. | 95% Confidence Interval | |
|  |  |  |  |  |  | Lower Bound | Upper Bound |
| Intercept | 3.698833 | .203198 | 180 | 18.203 | .000 | 3.297877 | 4.099789 |
| [Group=1] | .379272 | .136244 | 180 | 2.784 | .006 | .110432 | .648112 |
| [Group=2] | 0^b^ | 0 | . | . | . | . | . |
| [Specialty=1] | -.169689 | .207876 | 180 | -.816 | .415 | -.579875 | .240498 |
| [Specialty=2] | -.263509 | .229513 | 180 | -1.148 | .252 | -.716390 | .189373 |
| [Specialty=3] | .412744 | .210705 | 180 | 1.959 | .052 | -.003025 | .828514 |
| [Specialty=4] | 0^b^ | 0 | . | . | . | . | . |
| [Grade=1] | .695432 | .262308 | 180 | 2.651 | .009 | .177838 | 1.213027 |
| [Grade=2] | .034831 | .204916 | 180 | .170 | .865 | -.369516 | .439177 |
| [Grade=4] | 0^b^ | 0 | . | . | . | . | . |
| a. Dependent Variable: VigourT2. | | | | | | | |
| b. This parameter is set to zero because it is redundant. | | | | | | | |

**Comment**: There are highly significant differences in Vigour at T2 for all three factors (p=0.006 for Group, p=0.004 for Specialty and p= 0.003 for Grade, Table o) From table p, PW mean T2 Vigour score is 0.379 higher than controls, Rehab has highest score among Specialties (with Elderly being next highest) and Nurse Manager has (by a considerable margin) highest score among Grades.

**Mixed model results for T2**

**Absorption at T2**

| **Table q: Type III Tests of Fixed Effects^a^** | | | | |
| --- | --- | --- | --- | --- |
| Source | Numerator df | Denominator df | F | Sig. |
| Intercept | 1 | 180 | 2219.104 | .000 |
| Group | 1 | 180 | .326 | .569 |
| Specialty | 3 | 180 | 3.401 | .019 |
| Grade | 2 | 180 | 1.805 | .167 |
| a. Dependent Variable: AbsorptionT2. | | | | |

| **Table r: Estimates of Fixed Effects^a^** | | | | | | | |
| --- | --- | --- | --- | --- | --- | --- | --- |
| Parameter | Estimate | Std. Error | df | t | Sig. | 95% Confidence Interval | |
|  |  |  |  |  |  | Lower Bound | Upper Bound |
| Intercept | 4.206189 | .210716 | 180 | 19.961 | .000 | 3.790397 | 4.621980 |
| [Group=1] | .080662 | .141285 | 180 | .571 | .569 | -.198125 | .359449 |
| [Group=2] | 0^b^ | 0 | . | . | . | . | . |
| [Specialty=1] | -.547537 | .215567 | 180 | -2.540 | .012 | -.972901 | -.122174 |
| [Specialty=2] | -.449946 | .238005 | 180 | -1.890 | .060 | -.919584 | .019692 |
| [Specialty=3] | -.053766 | .218501 | 180 | -.246 | .806 | -.484919 | .377387 |
| [Specialty=4] | 0^b^ | 0 | . | . | . | . | . |
| [Grade=1] | .474733 | .272013 | 180 | 1.745 | .083 | -.062012 | 1.011479 |
| [Grade=2] | .131185 | .212498 | 180 | .617 | .538 | -.288122 | .550493 |
| [Grade=4] | 0^b^ | 0 | . | . | . | . | . |
| a. Dependent Variable: AbsorptionT2. | | | | | | | |
| b. This parameter is set to zero because it is redundant. | | | | | | | |

**Comment:** Absorption scores at T2 differ significantly only by Specialty (p=0.019 Table q), with Specialty’s 3 and 4 (Rehab and Elderly) scoring substantially higher than the other two specialties (see column 2 of Table r). Mean absorption score is only marginally higher in the PW group compared with the control group.

**Mixed model results for T2**

**Dedication at T2**

| **Table s: Type III Tests of Fixed Effects^a^** | | | | |
| --- | --- | --- | --- | --- |
| Source | Numerator df | Denominator df | F | Sig. |
| Intercept | 1 | 180 | 2483.060 | .000 |
| Group | 1 | 180 | 1.781 | .184 |
| Specialty | 3 | 180 | 5.800 | .001 |
| Grade | 2 | 180 | 1.119 | .329 |
| a. Dependent Variable: DedicationT2. | | | | |

| **Table t: Estimates of Fixed Effects^a^** | | | | | | | |
| --- | --- | --- | --- | --- | --- | --- | --- |
| Parameter | Estimate | Std. Error | df | t | Sig. | 95% Confidence Interval | |
|  |  |  |  |  |  | Lower Bound | Upper Bound |
| Intercept | 4.441282 | .217729 | 180 | 20.398 | .000 | 4.011652 | 4.870912 |
| [Group=1] | .194850 | .145987 | 180 | 1.335 | .184 | -.093215 | .482916 |
| [Group=2] | 0^b^ | 0 | . | . | . | . | . |
| [Specialty=1] | -.391550 | .222741 | 180 | -1.758 | .080 | -.831070 | .047971 |
| [Specialty=2] | -.345368 | .245926 | 180 | -1.404 | .162 | -.830636 | .139900 |
| [Specialty=3] | .370554 | .225773 | 180 | 1.641 | .102 | -.074948 | .816056 |
| [Specialty=4] | 0^b^ | 0 | . | . | . | . | . |
| [Grade=1] | .343479 | .281066 | 180 | 1.222 | .223 | -.211130 | .898088 |
| [Grade=2] | .040124 | .219570 | 180 | .183 | .855 | -.393139 | .473387 |
| [Grade=4] | 0^b^ | 0 | . | . | . | . | . |
| a. Dependent Variable: DedicationT2. | | | | | | | |
| b. This parameter is set to zero because it is redundant. | | | | | | | |

**Comment:** Dedication scores differ significantly at T2 only by Specialty (p=0.001 Table s)and this score is substantially higher in the Rehab specialty compared with Elderly, which in turn is substantially higher compared with the other specialties (Column 2 of Table t).

**Summary for T2**

Specialty is significant for all engagement measures at T2, with Rehab (and sometimes Elderly) consistently showing higher scores than other two specialties. The PW group at T2 shows significantly higher scores only for Vigour.

**Mixed model results for changes in scores (T2 – T1)**

**Change in Total Engagement Score (TES)**

| **Table u: Type III Tests of Fixed Effects^a^** | | | | |
| --- | --- | --- | --- | --- |
| Source | Numerator df | Denominator df | F | Sig. |
| Intercept | 1 | 180 | .724 | .396 |
| Group | 1 | 180 | 1.079 | .300 |
| Specialty | 3 | 180 | 3.663 | .013 |
| Grade | 2 | 180 | .607 | .546 |
| a. Dependent Variable: Change in Total Engagement Score. | | | | |

| **Table v: Estimates of Fixed Effects^a^** | | | | | | | |
| --- | --- | --- | --- | --- | --- | --- | --- |
| Parameter | Estimate | Std. Error | df | t | Sig. | 95% Confidence Interval | |
|  |  |  |  |  |  | Lower Bound | Upper Bound |
| Intercept | -.192253 | .205527 | 180 | -.935 | .351 | -.597805 | .213299 |
| [Group=1] | -.143137 | .137805 | 180 | -1.039 | .300 | -.415059 | .128784 |
| [Group=2] | 0^b^ | 0 | . | . | . | . | . |
| [Specialty=1] | -.117142 | .210258 | 180 | -.557 | .578 | -.532030 | .297746 |
| [Specialty=2] | -.071638 | .232143 | 180 | -.309 | .758 | -.529710 | .386435 |
| [Specialty=3] | .449302 | .213120 | 180 | 2.108 | .036 | .028768 | .869837 |
| [Specialty=4] | 0^b^ | 0 | . | . | . | . | . |
| [Grade=1] | .150878 | .265315 | 180 | .569 | .570 | -.372649 | .674405 |
| [Grade=2] | .224043 | .207265 | 180 | 1.081 | .281 | -.184938 | .633025 |
| [Grade=4] | 0^b^ | 0 | . | . | . | . | . |
| a. Dependent Variable: Change in Total Engagement Score. | | | | | | | |
| b. This parameter is set to zero because it is redundant. | | | | | | | |

**Comment:** Table u shows that change in total score is significantly associated only with Specialty (P=0.013, p> 0.05 for group and Grade). Table v shows that Specialty 3 (Rehab) has significantly different (higher) change in total score than Specialty 4 (Elderly) (P=0.036), the reference Specialty, but that changes at other Specialties are not significantly different from Specialty 4.

**Mixed model results for changes in scores (T2 – T1)**

**Change in Vigour**

| **Table w: Type III Tests of Fixed Effects^a^** | | | | |
| --- | --- | --- | --- | --- |
| Source | Numerator df | Denominator df | F | Sig. |
| Intercept | 1 | 180 | .848 | .358 |
| Group | 1 | 180 | .064 | .801 |
| Specialty | 3 | 180 | 3.840 | .011 |
| Grade | 2 | 180 | .977 | .378 |
| a. Dependent Variable: Change in Vigour score. | | | | |

| **Table x: Estimates of Fixed Effects^a^** | | | | | | | |
| --- | --- | --- | --- | --- | --- | --- | --- |
| Parameter | Estimate | Std. Error | df | t | Sig. | 95% Confidence Interval | |
|  |  |  |  |  |  | Lower Bound | Upper Bound |
| Intercept | -.470220 | .246113 | 180 | -1.911 | .058 | -.955858 | .015418 |
| [Group=1] | -.041686 | .165018 | 180 | -.253 | .801 | -.367305 | .283933 |
| [Group=2] | 0^b^ | 0 | . | . | . | . | . |
| [Specialty=1] | -.008336 | .251779 | 180 | -.033 | .974 | -.505154 | .488482 |
| [Specialty=2] | .028645 | .277986 | 180 | .103 | .918 | -.519885 | .577174 |
| [Specialty=3] | .651161 | .255205 | 180 | 2.552 | .012 | .147581 | 1.154740 |
| [Specialty=4] | 0^b^ | 0 | . | . | . | . | . |
| [Grade=1] | .338868 | .317707 | 180 | 1.067 | .288 | -.288042 | .965778 |
| [Grade=2] | .344008 | .248194 | 180 | 1.386 | .167 | -.145736 | .833752 |
| [Grade=4] | 0^b^ | 0 | . | . | . | . | . |
| a. Dependent Variable: Change in Vigour score. | | | | | | | |
| b. This parameter is set to zero because it is redundant. | | | | | | | |

**Comment:** Change in Vigour score significantly different only by Specialty (p=0.011 Table w) and Rehab shows significantly higher change in Vigour score than Elderly (p=0.012, Table x) but other Specialty’s do not.

**Mixed model results for changes in scores (T2 – T1)**

**Change in Absorption**

| **Table y: Type III Tests of Fixed Effects^a^** | | | | |
| --- | --- | --- | --- | --- |
| Source | Numerator df | Denominator df | F | Sig. |
| Intercept | 1 | 180 | 2.518 | .114 |
| Group | 1 | 180 | 5.217 | .024 |
| Specialty | 3 | 180 | 1.050 | .372 |
| Grade | 2 | 180 | 1.072 | .344 |
| a. Dependent Variable: Change in Absorption score. | | | | |

| **Table z: Estimates of Fixed Effects^a^** | | | | | | | |
| --- | --- | --- | --- | --- | --- | --- | --- |
| Parameter | Estimate | Std. Error | df | t | Sig. | 95% Confidence Interval | |
|  |  |  |  |  |  | Lower Bound | Upper Bound |
| Intercept | .343182 | .236708 | 180 | 1.450 | .149 | -.123896 | .810261 |
| [Group=1] | -.362508 | .158712 | 180 | -2.284 | .024 | -.675683 | -.049332 |
| [Group=2] | 0^b^ | 0 | . | . | . | . | . |
| [Specialty=1] | -.167904 | .242157 | 180 | -.693 | .489 | -.645736 | .309927 |
| [Specialty=2] | -.032964 | .267362 | 180 | -.123 | .902 | -.560531 | .494603 |
| [Specialty=3] | .201533 | .245453 | 180 | .821 | .413 | -.282801 | .685868 |
| [Specialty=4] | 0^b^ | 0 | . | . | . | . | . |
| [Grade=1] | -.164999 | .305566 | 180 | -.540 | .590 | -.767951 | .437952 |
| [Grade=2] | .153874 | .238709 | 180 | .645 | .520 | -.317154 | .624902 |
| [Grade=4] | 0^b^ | 0 | . | . | . | . | . |
| a. Dependent Variable: Change in Absorption score. | | | | | | | |
| b. This parameter is set to zero because it is redundant. | | | | | | | |

**Comment:**  Change in Absorption differs significantly only by Group (p=0.024 Table y) and this is due to an average change of-0.362508) in PW group compared with controls (p=0.024 Table 2). That is, control group did better here.

**Mixed model results for changes in scores (T2 – T1)**

**Change in Dedication**

| **Table z1: Type III Tests of Fixed Effects^a^** | | | | |
| --- | --- | --- | --- | --- |
| Source | Numerator df | Denominator df | F | Sig. |
| Intercept | 1 | 180 | .424 | .516 |
| Group | 1 | 180 | 2.075 | .151 |
| Specialty | 3 | 180 | 2.015 | .113 |
| Grade | 2 | 180 | .061 | .941 |
| a. Dependent Variable: Change in Dedication score. | | | | |

| **Table z2: Estimates of Fixed Effects^a^** | | | | | | | |
| --- | --- | --- | --- | --- | --- | --- | --- |
| Parameter | Estimate | Std. Error | df | t | Sig. | 95% Confidence Interval | |
|  |  |  |  |  |  | Lower Bound | Upper Bound |
| Intercept | -.159088 | .240457 | 180 | -.662 | .509 | -.633565 | .315388 |
| [Group=1] | -.232230 | .161226 | 180 | -1.440 | .151 | -.550366 | .085906 |
| [Group=2] | 0^b^ | 0 | . | . | . | . | . |
| [Specialty=1] | .032037 | .245992 | 180 | .130 | .897 | -.453363 | .517436 |
| [Specialty=2] | .110542 | .271597 | 180 | .407 | .684 | -.425381 | .646465 |
| [Specialty=3] | .492322 | .249340 | 180 | 1.974 | .050 | .000316 | .984328 |
| [Specialty=4] | 0^b^ | 0 | . | . | . | . | . |
| [Grade=1] | .066741 | .310406 | 180 | .215 | .830 | -.545761 | .679243 |
| [Grade=2] | .084550 | .242490 | 180 | .349 | .728 | -.393939 | .563039 |
| [Grade=4] | 0^b^ | 0 | . | . | . | . | . |
| a. Dependent Variable: Change in Dedication score. | | | | | | | |
| b. This parameter is set to zero because it is redundant. | | | | | | | |

**Comment:** Change in Dedication was not significantly different by any of Group, Specialty or Grade (Table z1). However (p=0.05 Table z2) change in Dedication at Specialty 3 (Rehab) shows borderline significance compared with Specialty 4 (Elderly)
